# Synergistic effect of the amendment on soybean yield increase and soil remediation in soda saline–alkali land: focusing on the soil quality index

**DOI:** 10.3389/fpls.2026.1818215

**Published:** 2026-05-08

**Authors:** Mingfen Shan, Chen Wang, Yuxian Zhang, Zhongxia Yu, Yuxin Fan, Anni Bai, Lei Lu, Linfeng Ren, Yaokun Wu, Wei Zhou, Mingcong Zhang

**Affiliations:** 1 College of Agronomy, Heilongjiang Bayi Agricultural University, Daqing, China; 2 National Engineering Research Center for Cereals, Daqing, China; 3 Heilongjiang Academy of Sciences, Daqing, China; 4 Daqing Qilong Agricultural Science and Technology Limited Company, Daqing, China; 5 Key Laboratory of Low-carbon Green Agriculture in Northeastern China, Ministry of Agriculture and Rural Affairs P. R. China, Daqing, China

**Keywords:** soda saline–alkali soil, soil amendment, soil quality index, soybean yield, water-soluble anion, water-soluble cation

## Abstract

Understanding the relationship between the application of soil conditioners and soil quality is crucial for achieving sustainably high crop yields and the remediation of saline-alkali soils in agricultural systems. However, to date, there have been few reports on the use of soil quality index (SQI) to characterize soil quality restoration in different soil layers of saline alkali land. We conducted a field experiment over three growing seasons (2022 – 2024) and collected soil samples after soybean harvest in 2024. The following treatments were employed: conventional fertilization (CK), conventional fertilization plus 1 t ha^-^¹ citric acid (T1), conventional fertilization plus 1 t ha^-^¹ biochar (T2), and conventional fertilization plus 1.5 t ha^-^¹ organic fertilizer (T3). Based on the Soil Quality Index, a comprehensive evaluation was performed to assess variations in soil fertility, alkalization index, and crop yield across three soil depths: 0–10 cm, 10–20 cm, and 20–30 cm. The research results indicate that: Compared with the CK treatment, the application of amendments significantly increased the concentrations of available nitrogen, available phosphorus, and available potassium. Conversely, it reduced soil pH (0.95%-5.83%) and decreased levels of exchangeable sodium, exchangeable sodium percentage (10.88%-30.12%), and sodium ion (20.26%-37.15%). The amendments significantly enhanced the SQI through synergistic regulation of soil fertility and salinity. Specifically, the citric acid treatment (T1) yielded the most pronounced improvement in the 0–10 cm soil layer, whereas the organic fertilizer amendment (T3) was more effective at depths of 10–30 cm. The soybean yield increase effect of citric acid treatment (T1) is the best, with a yield increase rate of 42.67% (*P* < 0.05). In summary, citric acid (T1) optimizes nutrient availability by alleviating saline-alkali stress, laying a theoretical foundation for ecological restoration in alkaline soils and enhancing agricultural productivity.

## Graphical Abstract

**Figure:**
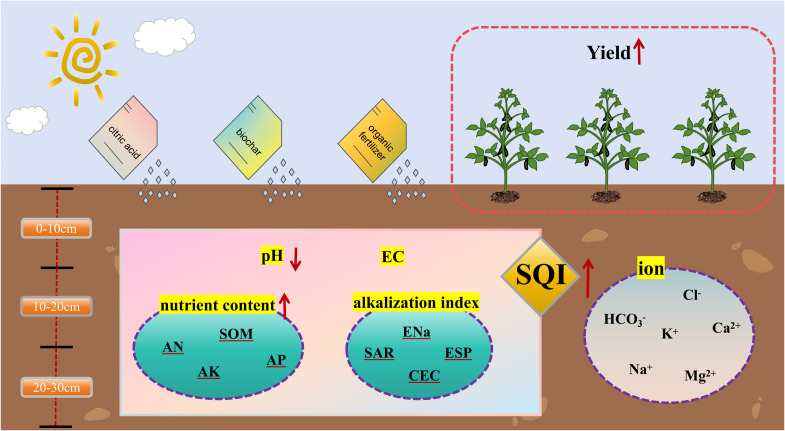

## 1 Introduction

Soda saline-alkali soil, as one of the globally widespread types of problematic soils, covers an area of 83 to 93 million hectares worldwide; the Songnen Plain represents the primary distribution area of soda saline-alkali soils in China, encompassing a total affected area of 3.7×10^6^ hm² (Yang et al., 2022; Liu et al., 2023). The surface layer of these soils is characterized by high salinity and severe saline-alkali limitations (Huang et al., 2025b). This leads to the disintegration of soil structure, reduced a reduction in nutrient availability, and elevated sodium ion toxicity, thereby severely compromising soil quality and function (Huang et al., 2025a). Current remediation strategies for soda saline-alkali soils, both domestically and internationally, frequently prioritize the short-term alleviation of specific constraints. A comprehensive, quantitative assessment of overall soil quality restoration remains notably absent. This limitation leads to transient improvements and hinders the synergistic optimization of two critical objectives: restoring soil ecological functions and achieving agricultural production goals (Yang et al., 2024). Consequently, the SQI, which integrates physical, chemical, and biological indicators, has emerged as a central tool in evaluating saline-alkali soil remediation, offering systematic, sensitive, and predictive advantages. Domestic and international research has established comprehensive soil quality assessment as a fundamental breakthrough in the management of saline-alkali soil (Xiao et al., 2024). The soil health assessment framework developed by the United States Department of Agriculture (USDA) (Ozlu et al., 2022) and the multiscale soil function model proposed by the European Union (EU) (Hemati et al., 2020) both underscore the critical importance of the SQI as a tool for systematic integration. The soil quality grading standards proposed by Sun Bo (Bhuyan et al., 2024) and the SQI model for saline soil developed by Zhang Jiabao (Yu et al., 2018) have both confirmed the effectiveness of quantifying the soil remediation process, laying the foundation for the technical paradigm of “multi index integration standardization principal component analysis weighting”.

In the research of saline alkali land improvement technology, various measures such as physics, chemistry, biology, and biochemistry have been widely explored and applied (Hou et al., 2025; Fan et al., 2025; Zhang et al., 2019). Applying soil amendments is a common practice for improving the overall soil environment in saline alkali soils (Sun et al., 2021). Among them, citric acid, as a representative substance of low molecular weight organic acids, has a strong chelating ability due to the active carboxyl functional group (-COOH) in its molecular structure (Li et al., 2021). Utilizing its intramolecular coordination sites, citric acid chelates with sodium ions adsorbed onto soil colloid surfaces, thereby displacing them and facilitating their leaching and removal (Hu et al., 2021). Biochar, as a carbonaceous material with highly porous structure and abundant surface negative charge, has shown unique advantages in the physical improvement of saline alkali soil (Zhang et al., 2025). It can function as a binder, facilitating soil particle aggregation into stable structures that enhance hydraulic conductivity and soil aeration (Wu et al., 2025). At the same time, the surface of biochar is rich in negative charges (Wang et al., 2023), which can adsorb base ions through electrostatic attraction and reduce the ion strength of soil solutions (Lee et al., 2021; Sun et al., 2021). The important role of organic fertilizers in the fertilization and improvement of saline alkali soil is increasingly being recognized (Liu et al., 2024). The activated carbon produced by its decomposition can drive microorganisms to synthesize polymer salt absorbents, thereby reducing the concentration of Na^+^ and Cl^-^ ions in saline-alkali soils (Gu et al., 2022). Prevalent in organic fertilizers, humic substances feature oxygen-containing functional groups (including carboxyl and phenolic hydroxyl groups) that dissociate into negatively charged sites. These sites electrostatically attract Na^+^, leading to ion-exchange adsorption and thereby mitigating sodium toxicity in the soil (Wang et al., 2023). These mechanisms collectively enhance soil structural stability, ameliorate sodium toxicity stress, and promote microbial-driven functions. Consequently, they contribute to the substantial optimization of key parameters across the physical, chemical, and biological dimensions of the SQI.

Early soil quality assessment often used mixed soil layer samples (Gao et al., 2025; Song et al., 2025), and there was relatively insufficient research on the differentiation of soil characteristics between different soil layers. The formation of crop productivity is regulated by the comprehensive characteristics of multiple soil layers within the entire soil profile. Although the evaluation method based on a single soil layer has the advantages of convenient sampling and efficient measurement, there are obvious limitations to the soil functional information it can reflect. Therefore, establishing a comprehensive evaluation index system covering different soil layers for soil quality assessment can help more accurately identify the dominant limiting factors that constrain key soil functions.

In order to fill these knowledge gaps, a field experiment was conducted in 2022 to evaluate the effects of different amendments (citric acid, biochar, organic fertilizer) on soil quality index and soybean yield in the 0 – 10, 10 – 20, and 20–30 cm soil layers of soda saline alkali soil in the Songnen Plain. The research objectives include: i) analyzing the changes in soil nutrient content and alkalization indicators in different soil layers under the regulation of different amendments. ii) explore the relationship between soil indicators of different soil layers, soil quality index, and soybean yield. iii) use soil quality index to reveal the quantitative relationship between soil environmental factors and yield in farmland. Based on this, we propose the following hypothesis: i) amendments can improve soil quality by reducing soil salinity and increasing soil nutrient content. ii) the SQI in the 10 – 20cm soil layer has the most significant impact on soybean yield. Provide technical support for improving soil quality, precise improvement of saline alkali land, and increasing production capacity in soda saline alkali farmland.

## 2 Materials and methods

### 2.1 Description of the study site

**Figure 1:**
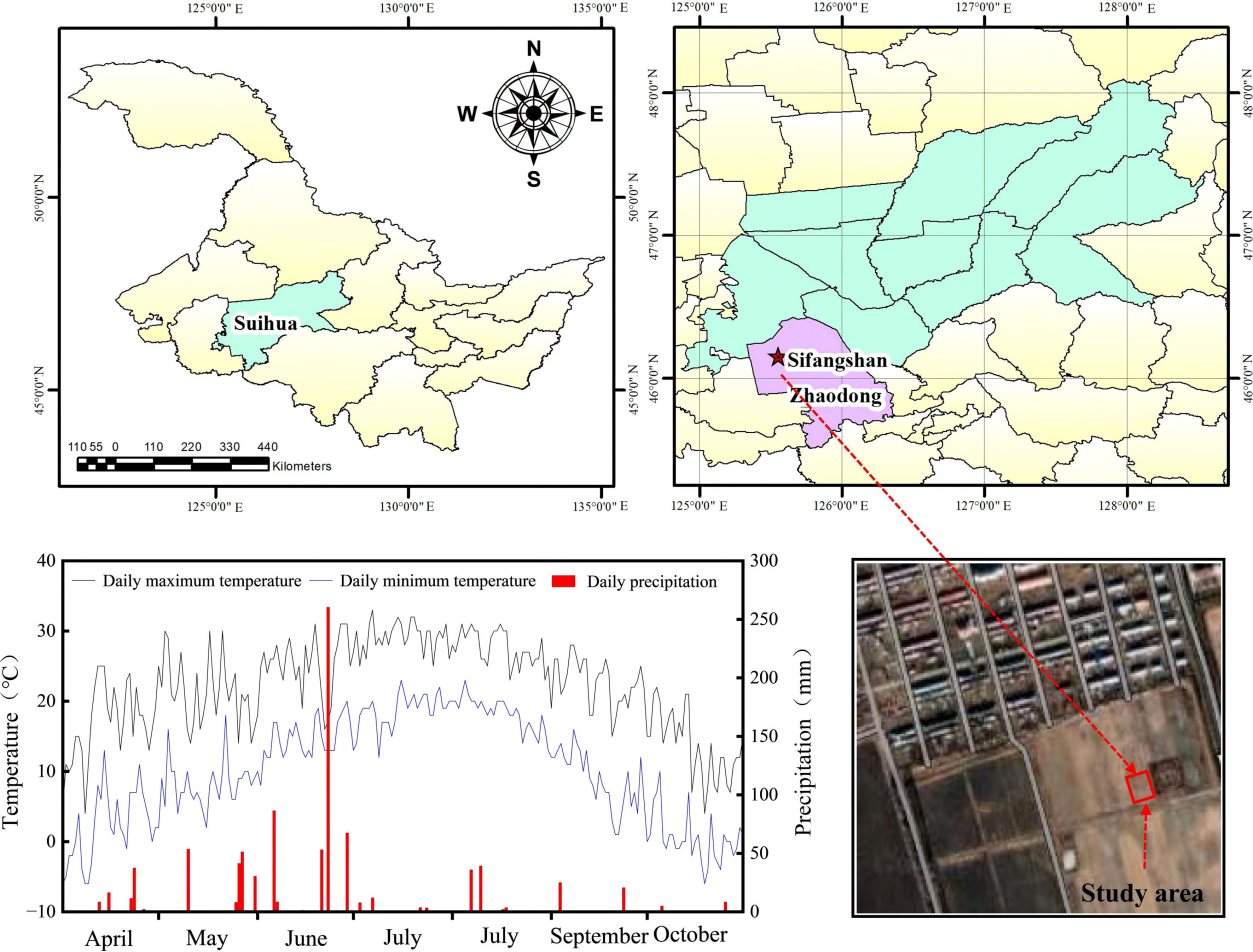
Sifangshan farm of the beidahuang group co., Ltd.

This study was conducted in 2022 at the Sifangshan Farm, operated by the Beidahuang Group Co., Ltd., using an annual corn-soybean rotation system. Plant soybeans in 2022, corn in 2023, and soybeans in 2024, during which key agronomic indicators were measured. The experimental site was situated in the central Songnen Plain, which features a cold-temperate continental monsoon climate with an annual average temperature of 3.1 °C. The four seasons are clearly differentiated, characterized by cold, warm, dry, and wet conditions. The frost-free period spans approximately 136 days annually, while the frozen period persists for approximately six months. The primary wind direction is northwest, although it exhibits considerable seasonal variation. **Figure 1** presents the daily maximum and minimum temperatures, as well as precipitation levels, recorded throughout the experimental period. The soil profile at a depth of 0–30 cm is characterized as soda saline–alkali soil, containing 12.2 g/kg of organic matter,78.5 mg/kg of available nitrogen, 196.7 mg/kg of available potassium, 25.06 mg/kg of available phosphorus, 9.1 of pH, and 382.3 μS/cm of EC.

### 2.2 Test materials

The test soybeans: soybean cultivar Qinong No. 5, branching, white flowers, pointed leaves, and gray pubescence. Its pods are sickle-shaped and turn brown at maturity.

The soil amendment: citric acid (Henan Shengkun Chemical Products Co., Ltd.); corn stover-derived biochar (Mishan City, Heilongjiang Province). The specific composition is shown in **Table 1**. Organic fertilizer (Heilongjiang Hongtai Bioorganic Fertilizer Co., Ltd.). The specific composition is shown in **Table 2**.

**Table 1:** Biochar composition table.

Specific surface area(m^2^·g^-1^)	Ash content	pH	C	N	P	K
7.29	27.90%	8.46	55.31%	1.35%	0.56%	1.42%

**Table 2:** Organic fertilizer composition table.

Total nutrients	Organic matter	N	P_2_O_5_	K_2_O	Net content
≥ 5%	≥ 30%	0.84%	1.85%	0.96%	50.0 ± 0.5 kg

### 2.3 Experimental design and methods

The experiment utilized a completely randomized design comprising four treatments: conventional fertilization (CK), conventional fertilization plus 1 t ha^-^¹ citric acid (T1), conventional fertilization plus 1 t ha^-^¹ biochar (T2), and conventional fertilization plus 1.5 t ha^-^¹ organic fertilizer (T3). The application rate for each treatment was based on the optimal rates determined in prior experimental phases. Each treatment was repeated three times. The experimental plots, each covering an area of 55.6 m², consisted of six ridges measuring 7 m in length and 1.3 m in width, and were separated by a 1 m buffer zone. The amendment is applied to the planting rows before soil preparation each autumn, and then plowed together with the 0–20 cm soil, ensuring thorough mixing. The crop was sown in the first week of May 2024 and harvested during the final week of September. All other agronomic practices followed conventional local protocols. During the process of mechanical ridge formation in autumn, fertilizer is buried in the soil. Urea (N ≥ 46%), diammonium phosphate (N ≥ 18%, P_2_O_5_ ≥ 46%), and potassium magnesium sulfate (K_2_O ≥ 24%, Mg ≥ 6%). Nitrogen, phosphorus, and potassium fertilizers were administered in a single basal application. Concurrently with sowing in the spring, ammonium sulfate was supplied at 95 kg/hm² (N ≥ 21%).

### 2.4 Test items and methods

#### 2.4.1 Soil and plant sample collection and analysis

In our case, soil at 0–10 cm, 10–20 cm, and 20–30 cm depths was sampled using a soil drill following the harvest of soybean in 2024. Sampled soil was packed in sealed plastic bags with labeled tags and transferred to the Lab. The soil samples were air-dried and ground. A pH meter (Lei Zhi PHBJ-261 L) and an EC meter (Lei Zhi DDBJ-351 L) were used to measure the soil pH and EC, respectively, at a ratio of 1:5 (soil: distilled water). Soil organic matter (SOM) content was quantified using the potassium dichromate oxidation method with external heating. Soil available nitrogen (AN) was determined by the alkali-hydrolyzed diffusion method, and soil available phosphate (AP)was measured by the molybdenum antimony colorimetric method. Soil available potassium (AK) was analyzed by the flame photometry method. The concentrations of water-soluble potassium ion (K^+^) and water-soluble sodium ion (Na^+^) were quantified using flame photometry, while water-soluble calcium ion (Ca²^+^) and water-soluble magnesium ion (Mg²^+^) were determined by atomic absorption spectrometry. Additionally, water-soluble carbonate ion (HCO_3_^-^) and water-soluble chloride ion (Cl^-^) were analyzed via indicator-based neutralization titration and silver nitrate titration, respectively. The exchangeable sodium content was measured via the ammonium acetate-ammonium hydroxide exchange method coupled with flame photometry, while the cation exchange capacity (CEC) was assessed using the sodium acetate exchange method (Bao, 2000). The exchangeable sodium percentage (ESP) (Equation 1) and the sodium adsorption ratio (SAR) (Equation 2) are defined by the following equations:

(1) ESP=exchangeable sodium/CEC×100%

(2) $$\text{SAR}=[Na^{+}]/\sqrt{([Ca^{2+}]+[Mg^{2+}])/2}$$

During the soybean harvest period, select representative 1 m^2^ soybean plants, repeat 3 times, thresh and dry them, and measure their yield. At the same time, 30 plants with consistent growth were randomly selected for seed testing in each treatment, and their plant height, stem thickness, number of pods per plant, number of grains per plant, and hundred grain weight were measured.

#### 2.4.2 Soil quality assessment

Soil quality was comprehensively assessed using the SQI, according to the methodology established by Masto et al. (2008). The raw data in this study were first normalized, followed by standardization using a linear membership function. Positive indicators—such as SOM, AN, AP, AK, and CEC—were evaluated using a function where higher values correspond to better quality (Equation 3). Conversely, negative indicators—including soil pH, EC, exchangeable sodium percentage, ESP, and SAR, were assessed using a function where lower values are more desirable (Equation 4). The linear transformation standardizes all indicators to a [0, 1] scale, thus facilitating direct comparison among measures with disparate units and dimensions. The weighting for each soil quality indicator was assigned based on its communality, which was derived from principal component analysis (Equation 5). The SQI was ultimately computed using a linear membership function and assigned weights (Equation 6). Higher SQI values denote superior soil quality, whereas lower values indicate degraded conditions. The equation is defined as follows:

(3) $${\text{S}}_{\text{i}}=(X-X_{min})/(X_{max}-X_{min})\;$$

(4) $${\text{S}}_{\text{i}}=(X_{max}-X)/(X_{max}-X_{min})\;$$

(5) $${\text{W}}_{\text{i}}=C_{i}/\sum_{i=1}^{n}C_{i}\;$$

(6) $$\text{SQI}=\sum_{i=1}^{n}W_{i}S_{i}\;$$

where S_i_ is 0–1 and is the linear score of the soil indicator, X is the soil variable content, X_min_ is the minimum soil variable content, X_max_ is the maximum soil variable content, *C*_i_ is the indicator score (linear or nonlinear), and *W*_i_ is the weighting value of soil indicators.

### 2.5 Statistical analysis

Data were collated and preliminarily processed using Microsoft Excel 2019. Statistical analyses, including one-way analysis of variance (ANOVA) and Duncan’s multiple range test (α = 0.05), were performed using SPSS 27.0 to determine significant differences among treatments [conventional fertilization (CK), conventional fertilization plus 1 t ha^-^¹ citric acid (T1), conventional fertilization plus 1 t ha^-^¹ biochar (T2), and conventional fertilization plus 1.5 t ha^-^¹ organic fertilizer (T3)]. Figures were generated using Origin 2024. Redundancy analysis (RDA) was conducted in Canoco 5 to examine the relationships between 0-10cm, 10-20cm and 20-30cm soil indicators and soybean yield parameters. Mantel tests were visualized via heatmaps to examine the correlations between soil quality indices and environmental factors (Soil pH; soil EC; organic matter; available nitrogen; available phosphorus; available potassium; water-soluble potassium ion; water-soluble sodium ion; water-soluble calcium ion; water-soluble magnesium ion; water-soluble carbonate ion; water-soluble chloride ion; exchangeable sodium; cation exchange capacity; exchangeable sodium percentage; sodium adsorption ratio). Use R language (“ggcor, plspm” package) to analyze causal relationships between variables.

## 3 Results and analysis

### 3.1 Fundamental physicochemical properties of the soil

**Figure 2:**
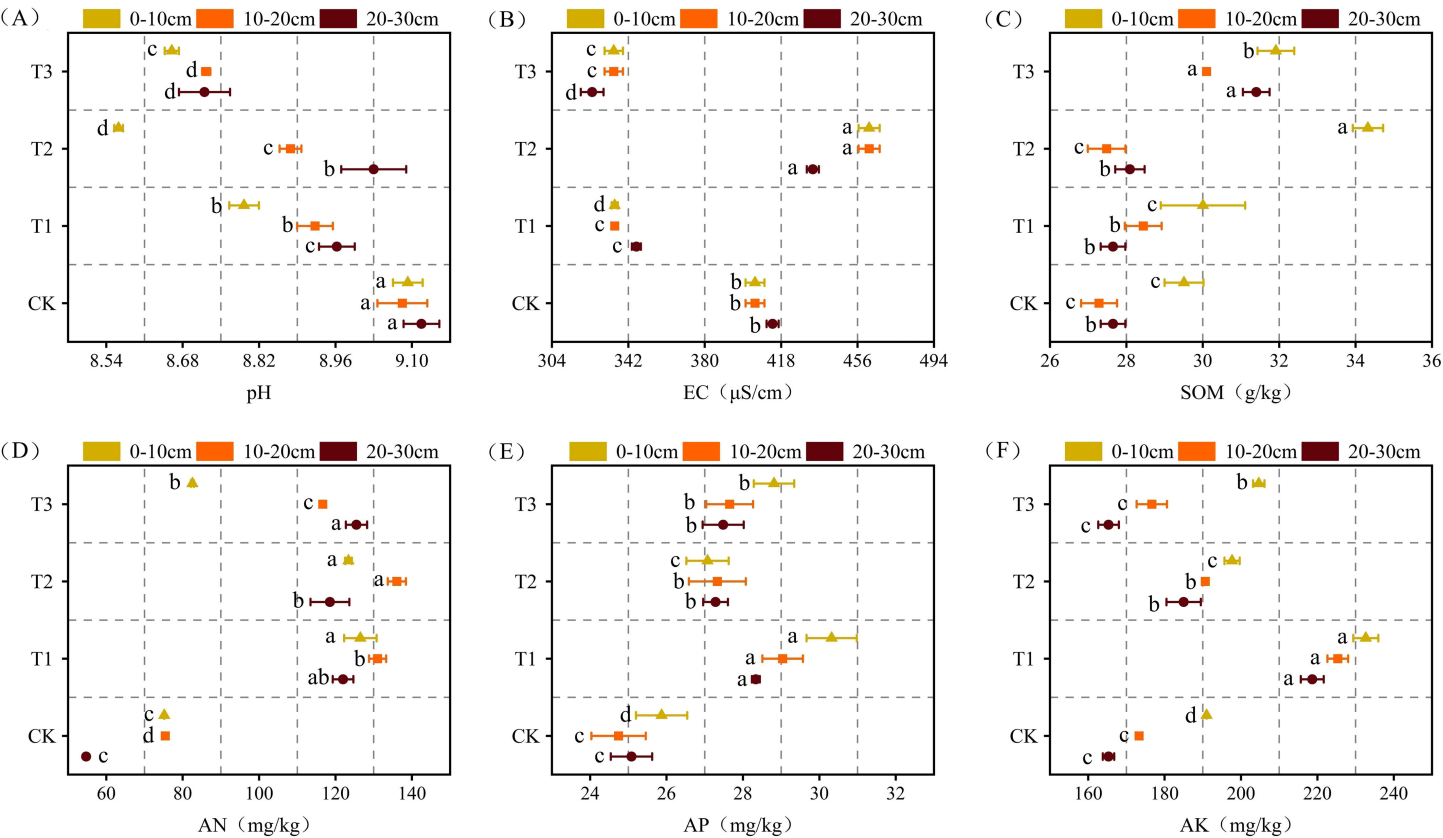
Basic physical and chemical properties of the soil under the different treatments. **(a)** pH, soil pH; **(b)** EC, soil EC; **(c)** SOM, organic matter; **(d)** AN, available Nitrogen; **(e)** AP, available phosphorus; **(f)** AK, available potassium. Different letters indicate a significant difference between the treatments at *P* < 0.05. Values represent mean ± standard error (n=3).

In comparison to all other treatments, T3 resulted in significantly lower soil pH and EC values across all soil layers (**Figures 2A, B**, *P* < 0.05). Soil pH varied significantly among the treatments. Compared with CK treatment, TI treatment significantly reduced pH by 1.68%~3.30%, and T2 treatment significantly reduced pH by 0.95%~5.83%. Among all treatments, T2 exhibited the highest EC values throughout the soil profile, with levels significantly greater than those in the CK treatment by 4.83% to 13.99%. The EC of each soil layer in T1 and T2 treatments was significantly lower than that in CK treatment. In the 0–10 cm soil layer, the T2 treatment exhibited the highest SOM content, which was 16.32% higher than that in CK treatment (**Figure 2C**, *P* < 0.05). In the 10–20 and 20–30 cm layers, SOM contents under the T3 treatment were significantly higher than those in all other treatments, showing an increase of 10.32% to 13.56% relative to the CK treatment. The AN content of the soil varied significantly both between treatments and across the soil layers (**Figure 2D**, *P* < 0.05). Compared with CK, the T1 treatment significantly increased AN content by 68.26% in the 0–10 cm layer, while the T2 treatment achieved an 80.29% increase in the 10–20 cm layer. Among all treatments, T1 resulted in the highest AP and AK contents across all soil layers (**Figures 2E, F**, *P* < 0.05). In comparison with CK, the AN content under the T1 treatment increased significantly by 12.97% to 17.35% across the various soil layers. The AK content in all treatments declined progressively with increasing soil depth.

### 3.2 Soil water-soluble cation and anion contents

**Figure 3:**
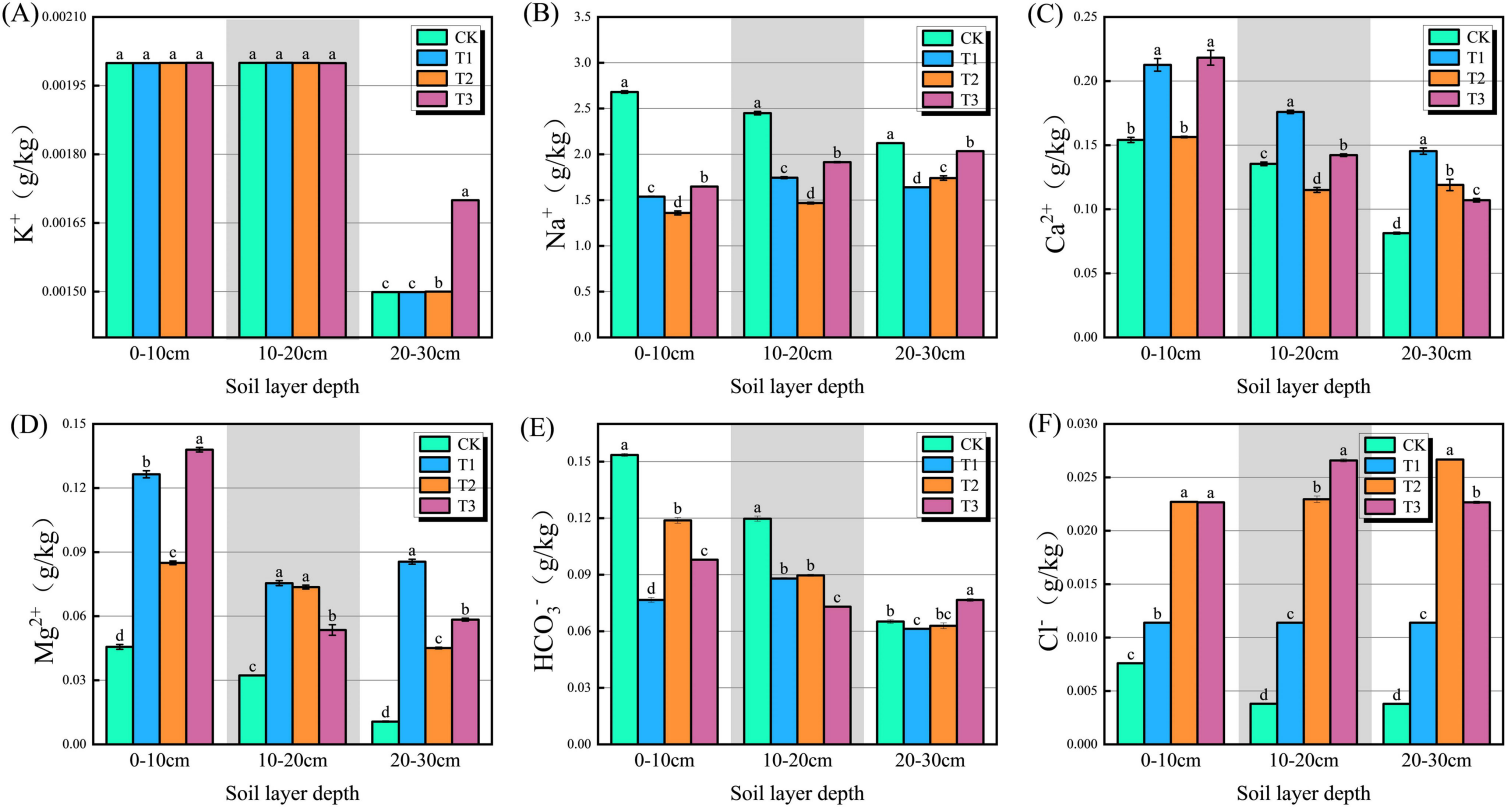
Contents of water-soluble anions and cations in soil under different treatments. **(a)** K^+^, water-soluble potassium ion; **(b)** Na^+^, water-soluble sodium ion; **(c)** Ca²^+^, water-soluble calcium ion; **(d)** Mg²^+^, water-soluble magnesium ion; **(e)** HCO_3_^-^, water-soluble carbonate ion; **(f)** Cl^-^, water-soluble chloride ion. Different letters indicate a significant difference between the treatments at *P* < 0.05. Values represent mean ± standard error (n=3).

The K^+^ content in the experimental soils decreased progressively with depth (**Figure 3A**, *P* < 0.05). In the 20–30 cm layer, the T2 and T3 treatments exhibited significant increases in water-soluble potassium content of 0.07% and 13.39%, respectively, compared to the CK treatment. In the 0–10 and 10–20 cm soil layers, the Na^+^ content across treatments decreased in the order: CK > T3 > T1 > T2 (**Figure 3B**, *P* < 0.05). In the 20–30 cm soil layer, water-soluble sodium content was significantly reduced in treatments T1, T2, and T3 relative to CK, with decreases of 22.75%, 17.98%, and 4.15%, respectively. The Ca²^+^ content generally decreased with soil depth in the CK, T1, and T3 treatments. In the 0–10 cm soil layer, Ca²^+^ content under the T1 and T3 treatments was significantly higher than that in CK, showing increases of 38.04% and 41.65%, respectively (**Figure 3C**, *P* < 0.05). In contrast, the T2 treatment decreased the Ca²^+^ content in the 10–20 cm layer by 15.04% compared to CK. Among all treatments, the 0–10 cm soil layer exhibited the highest Mg²^+^ content, with the T3 treatment showing a significantly greater concentration than the others (**Figure 3D**, *P* < 0.05). In the 10–20 cm layer, the Mg²^+^ concentrations in the T1, T2, and T3 treatments were significantly higher than those in the CK treatment. Conversely, in the 20–30 cm layer, the highest Mg²^+^ content was observed in the T1 treatment. The CK treatment exhibited significantly higher HCO_3_^-^ content in both the 0–10 cm and 10–20 cm soil layers compared with the other treatments (**Figure 3E**, *P* < 0.05). In the 20–30 cm layer, the HCO_3_^-^ content under the T3 treatment was 17.58% higher than that under the CK treatment, indicating a significant increase. The Cl^-^ content across all soil layers was significantly higher in the T1, T2, and T3 treatments than that in CK (**Figure 3F**, *P* < 0.05). In the 0–10 cm and 20–30 cm layers, soil Cl^-^ content was highest under the T2 treatment. In contrast, its concentration peaked in the 10–20 cm layer under the T3 treatment.

### 3.3 Soil alkalization index

**Figure 4:**
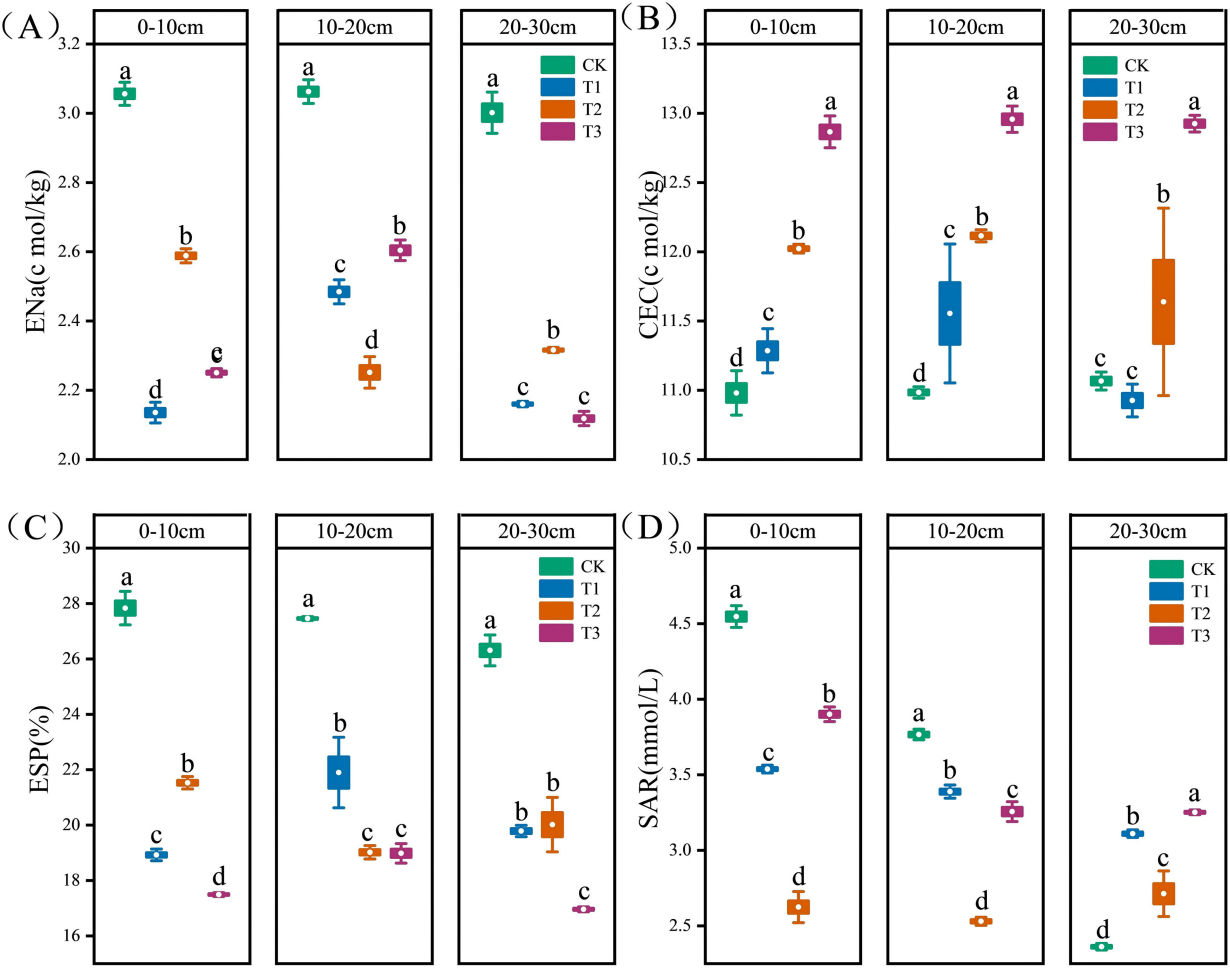
Soil alkalization indicators under different treatments. **(a)** ENa, Exchangeable sodium; **(b)** CEC, cation exchange capacity; **(c)** ESP, exchangeable sodium percentage; **(d)** SAR, sodium adsorption ratio. Different letters indicate a significant difference between the treatments at *P* < 0.05. Values represent mean ± standard error (n=3).

Across all soil layers, the exchangeable sodium content was highest in the CK treatment soil (**Figure 4A**, *P* < 0.05). In the 0–10 cm layer, the T1 treatment exhibited the lowest exchangeable sodium content, showing a significant reduction of 30.12% compared to CK. In the 10–20 cm layer, the T2 treatment yielded the lowest content, which was 19.25% lower than that in CK. Significant differences were observed among treatments in the 20–30 cm layer, although no significant difference was detected between T1 and T3. Across all soil layers, the T3 treatment exhibited the highest CEC, which was significantly greater than that of the CK treatment by 17.17%, 28.93%, and 13.50%, respectively (**Figure 4B**, *P* < 0.05). In the 0–10 and 10–20 cm layers, the CEC of the CK treatment was significantly lower than that of all amended treatments (T1, T2, T3). No significant difference in CEC was observed between the CK and T1 treatments in the 20–30 cm layer. The T2 treatment significantly increased CEC by 4.15% compared with the CK treatment. The ESP was reduced in the T1, T2, and T3 treatments relative to the CK, with decreases of 27.15%, 30.87%, and 35.53%, respectively (**Figure 4C**, *P* < 0.05). The T3 treatment showed the most substantial effect. In the 0–10 and 10–20 cm soil layers, the SAR was highest under the CK treatment and lowest under the T2 treatment (**Figure 4D**, *P* < 0.05). Compared to CK, the T2 treatment significantly reduced the ratio by 42.29% and 32.82% in these respective layers. In the 20–30 cm layer, the ratio was highest under the T3 treatment, which exhibited a significant increase of 37.66% compared to CK.

### 3.4 Soil quality index and soybean yield

**Figure 5:**
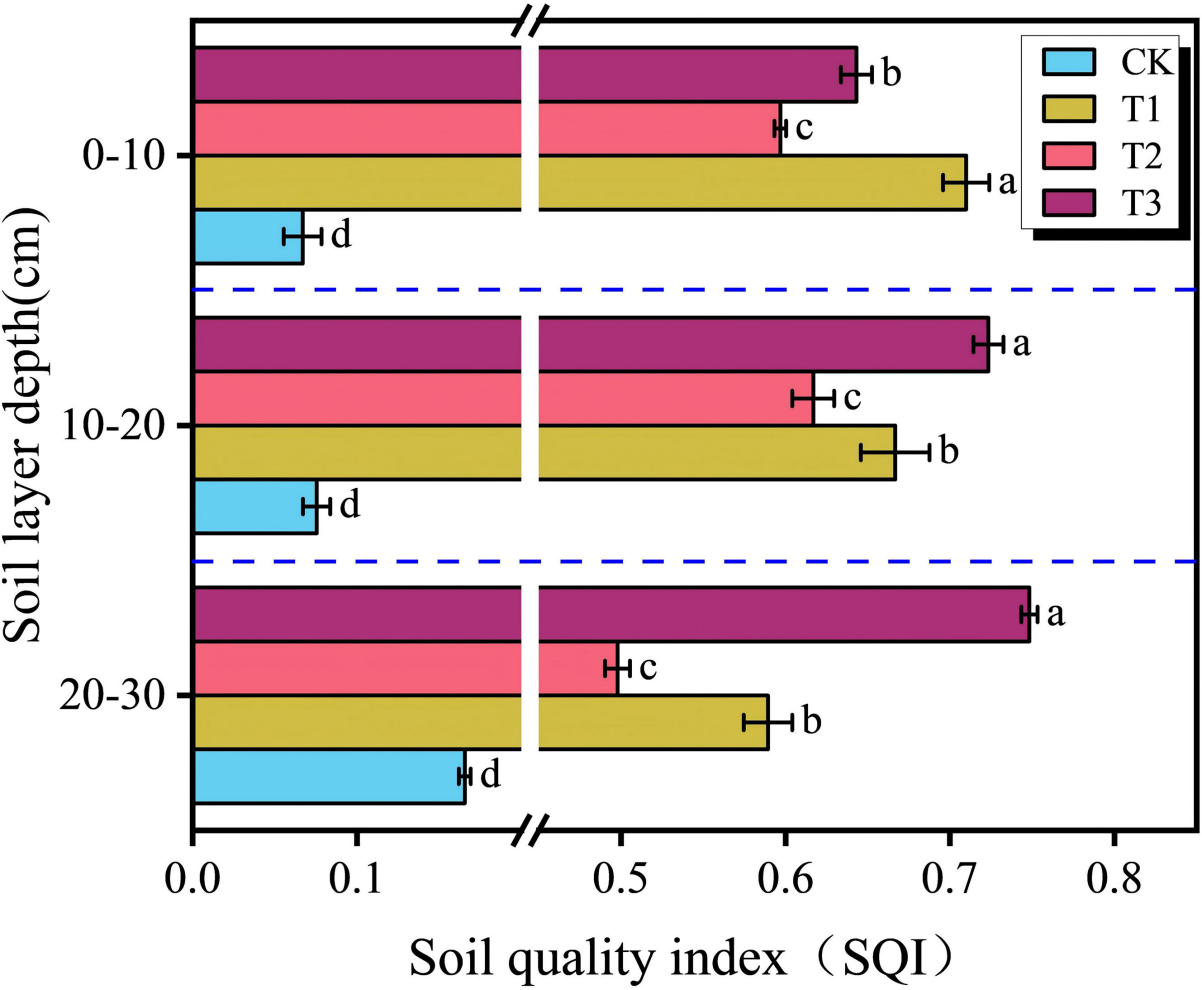
Soil quality indices under different treatments. Different letters indicate a significant difference between the treatments at *P* < 0.05. Values represent mean ± standard error (n=3).

The SQI was calculated for three soil depths (0 – 10, 10 – 20, and 20–30 cm) using a suite of ten indicators. The calculation involved principal component analysis, followed by the extraction of the communality for each indicator. Indicator weights were subsequently determined, enabling the final computation of the SQI (**Table 3**). Treatments T1, T2, and T3 significantly enhanced the SQI values across all soil depths relative to the CK (**Figure 5**, *P* < 0.05). In the 0–10 cm layer, SQI decreased in the order T1 > T3 > T2 > CK. In the deeper soil layers (10–20 and 20–30 cm), the order was T3 > T1 > T2 > CK.

**Table 3:** Common factor variance and weight of soil quality evaluation indicators.

Indicators	0-10cm		10-20cm		20-30cm	
	Communality	Weight	Communality	Weight	Communality	Weight
pH	0.999	0.101	0.969	0.098	0.934	0.102
EC	0.996	0.100	0.994	0.101	0.616	0.067
ENa	0.997	0.101	0.993	0.101	0.968	0.105
ESP	0.999	0.101	0.994	0.101	0.951	0.104
SAR	0.991	0.100	0.994	0.101	0.957	0.104
SOM	0.972	0.098	0.970	0.099	0.995	0.108
AN	0.998	0.101	0.997	0.101	0.912	0.099
AP	0.993	0.098	0.969	0.098	0.961	0.105
AK	0.994	0.100	0.992	0.101	0.967	0.105
CEC	0.994	0.100	0.971	0.099	0.925	0.101

**Figure 6:**
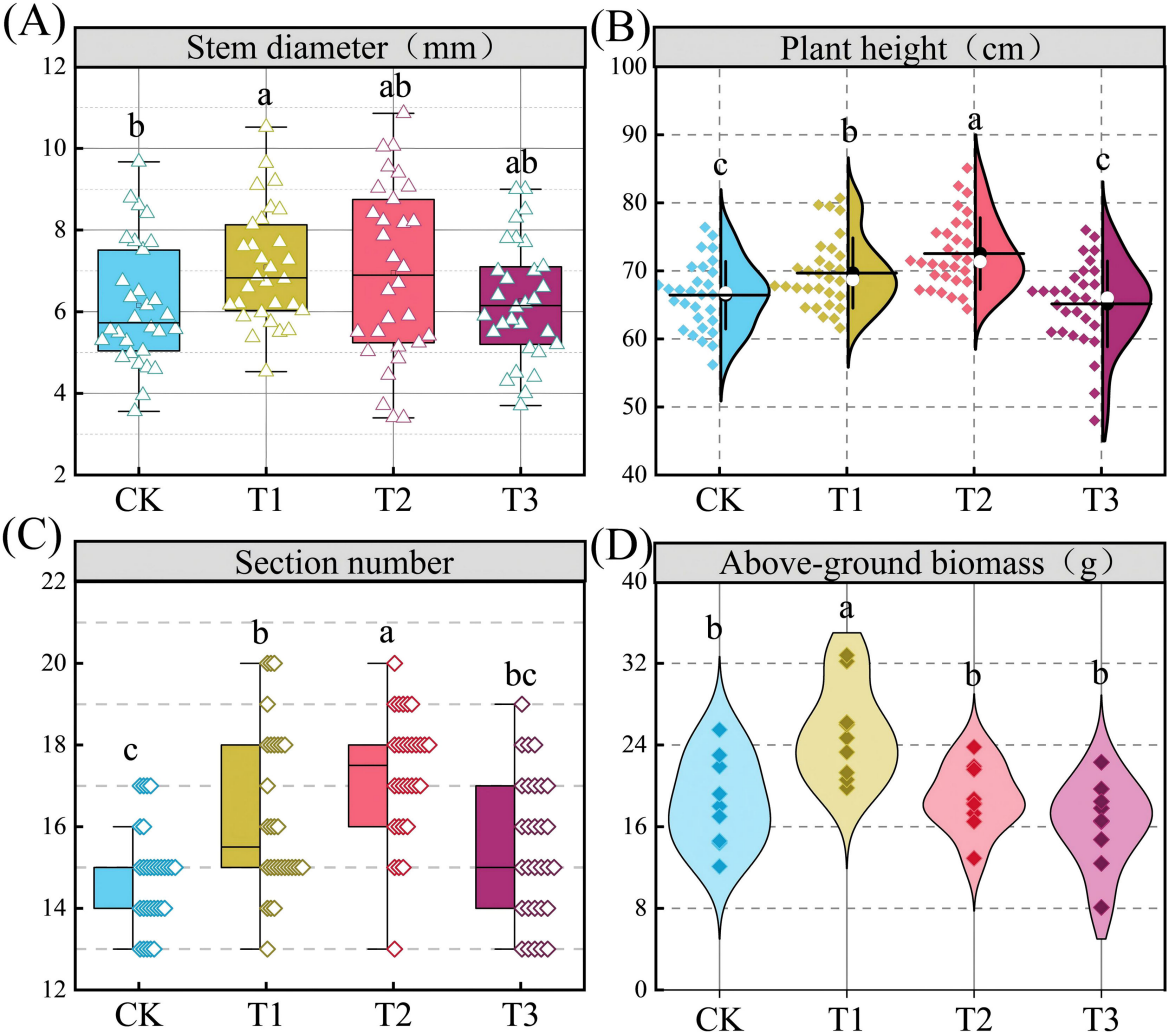
Plant biometric parameters. Different letters indicate a significant difference between the treatments at P < 0.05. The numerical values in **(a–c)** represent the mean ± standard error (n=30). The numerical values in **(d)** represent the mean ± standard error (n=9).

Compared with CK treatment, T1 and T2 treatments promoted the growth of stem diameter, plant height, and section number of nodes in the plants (**Figure 6**, *P* < 0.05). The above-ground biomass of T1 treatment was higher than that of other treatments. As shown in **Table 2** soybean yield varied significantly among the different treatments (*P* < 0.05). Relative to the CK treatment, soybean yields were significantly higher in the T1, T2, and T3 treatments (**Table 4**), showing increases of 57.0%, 28.2%, and 42.3%, respectively. Analysis of yield components further revealed that, compared to the control group (CK), the number of pods per plant increased by 77.39%, and the number of seeds per plant increased by 65.11%; these values were significantly higher than those observed in all other treatments. In contrast, the 100-seed weight was highest in the T3 treatment, with an increase of 69.08% compared to the CK treatment.

**Table 4:** Effects of different amendments on soybean yield and yield composition.

Treatment	Pods number per plant	Seeds number per plant	100-seed Weight/g	Yield/(kg·acre^-1^)
CK	28.00 ± 0.58ed	71.33 ± 0.67d	12.58 ± 0.12d	81.52 ± 1.20d
T1	49.67 ± 0.88a	121.67 ± 1.20a	16.91 ± 0.16c	127.96 ± 1.15a
T2	42.67 ± 0.88b	118.33 ± 1.45b	18.25 ± 0.15b	104.50 ± 1.59c
T3	32.67 ± 0.33c	88.33 ± 0.33c	21.27 ± 0.36a	115.96 ± 1.59b

The values are the means ± standard errors (n=3). Different letters indicate a significant difference between the treatments at *P* < 0.05.

**Figure 7:**
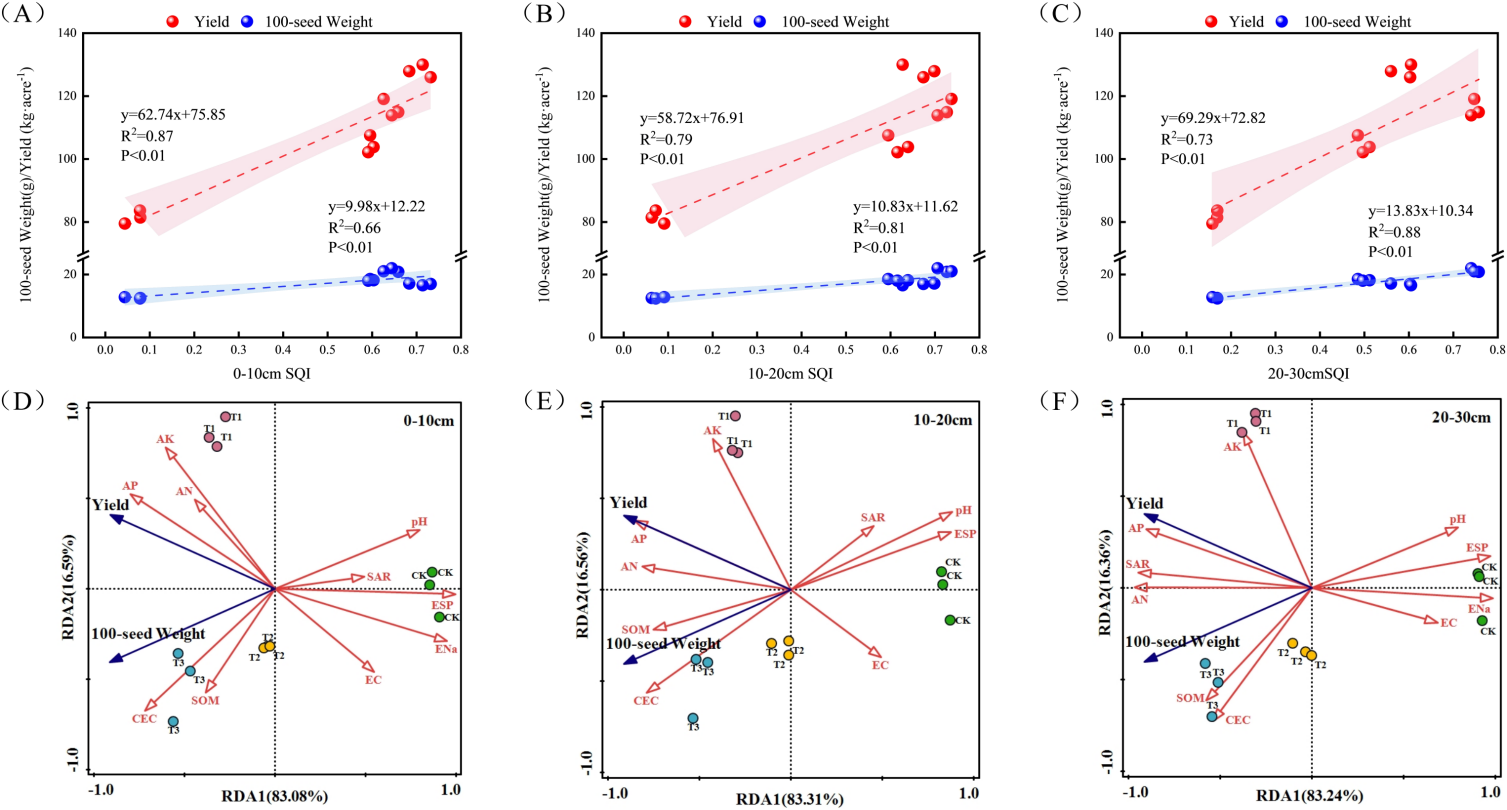
Linear fitting of soybean yield and the SQI. **(a)** Regression analysis of soybean yield and 100-seed weight at 0–10 cm SQI; **(b)** regression analysis of soybean yield and 100-seed weight at 10–20 cm SQI; **(c)** regression analysis of soybean yield and 100-seed weight at 20–30 cm SQI; **(d)** redundancy analysis between soybean yield and selected 0–10 cm soil parameters; **(e)** redundancy analysis between soybean yield and selected 10–20 cm soil parameters; **(f)** redundancy analysis between soybean yield and selected 20–30 cm soil parameters. pH, soil pH; EC, soil EC; SOM, organic matter; AN, available nitrogen; AP, available phosphorus; AK, available potassium; ENa, exchangeable sodium; CEC, cation exchange capacity; ESP, exchangeable sodium percentage; SAR, sodium adsorption ratio.

A linear regression analysis was performed to assess the relationships among soybean yield, 100-seed weight, and the SQI. Soybean yield exhibited the strongest correlation with the SQI in the 0–10 cm layer. Conversely, the 100-seed weight showed the strongest association with the soil quality index in the 20–30 cm layer. Notably, both yield and 100-seed weight were significantly positively correlated with the SQI across all measured soil layers (**Figures 7A–C**). RDA was conducted to elucidate the relationships between key soil properties (including pH, EC, SOM, AN, AP, AK, ENa, CEC, ESP, and SAR) and soybean yield components (**Figures 7D–F**). RDA showed that the first two axes (RDA1 and RDA2) accounted for a high cumulative proportion of the total variance at all soil depths: 99.67% (0–10 cm), 99.87% (10–20 cm), and 99.60% (20–30 cm). This result indicates that the selected soil indicators effectively explained the variability in soybean yield and 100-seed weight within each layer. Both soybean yield and 100-seed weight exhibited positive correlations with SOM, AN, AP, AK, and CEC across all soil layers. Conversely, these agronomic traits were negatively correlated with pH, EC, ENa, and ESP.

### 3.5 Comprehensive analysis

**Figure 8:**
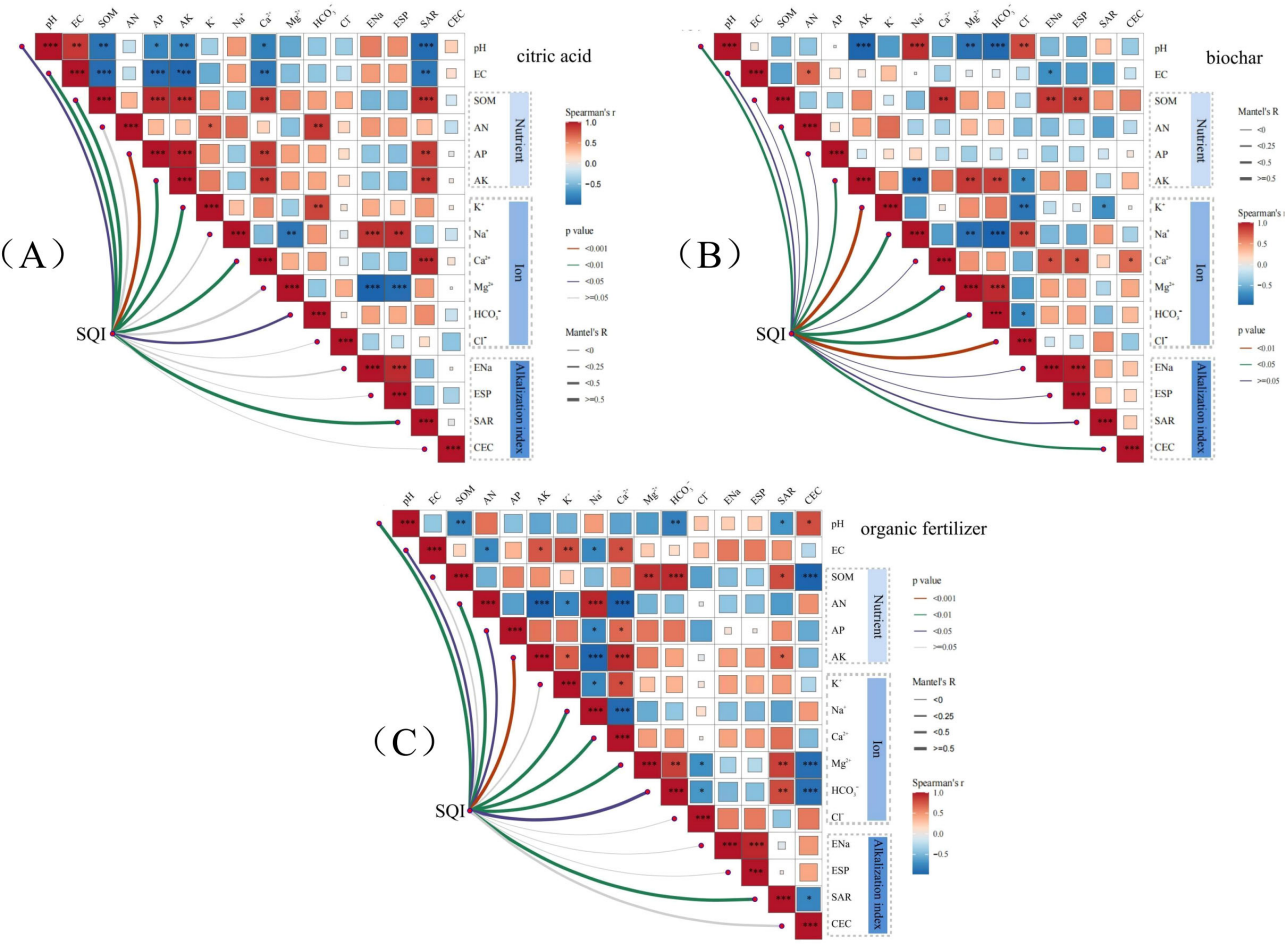
Correlation analysis between soybean yield and the SQI under the different amendments. **(a)** Citric acid; **(b)** biochar; **(c)** organic fertilizer. pH, Soil pH; EC, soil EC; SOM, organic matter; AN, available nitrogen; AP, available phosphorus; AK, available potassium; K^+^, water-soluble potassium ion; Na^+^, water-soluble sodium ion; Ca²^+^, water-soluble calcium ion; Mg²^+^, water-soluble magnesium ion; HCO_3_^-^, water-soluble carbonate ion; Cl^-^, water-soluble chloride ion; ENa, exchangeable sodium; CEC, cation exchange capacity; ESP, exchangeable sodium percentage; SAR, sodium adsorption ratio. In the figure, the color of each square denotes the sign (positive or negative) of the correlation coefficient between environmental factors. The thickness of the connecting lines corresponds to the strength of the correlation. *, **, and *** represent P < 0.05, P < 0.01, and P < 0.001, respectively.

This study employed the Mantel test in conjunction with heatmap analysis to identify the primary factors influencing the SQI following the application of soil amendments to saline-alkali soil (**Figure 8**). The distinct soil amendments produced markedly different correlation patterns between the SQI and measured soil properties. The addition of citric acid exhibited strong positive correlations with several soil parameters (**Figure 8A**, *P* < 0.001). It was significantly correlated with AP, AK, and SOM. Furthermore, it showed strong associations with ESP, Na^+^, and ENa. Correlations were also observed with Ca^2+^ and SAR. A strong negative correlation was observed between pH and SAR. Similarly, SOM, AP, AK, and EC showed strong negative correlations. ENa, ESP, and Mg^2+^ also exhibited significant negative correlations. Key soil properties, including pH, EC, SOM, AP, AK, K^+^, Ca^2+^, HCO_3_^-^, and SAR, showed strong and significant correlations with the SQI (r < 0.25, *P* < 0.05).

The biochar amendment resulted in significant positive correlations between Na^+^ and pH, HCO_3_^-^ and Mg²^+^, and ESP and ENa (**Figure 8B**, *P* < 0.001). In contrast, both AK and HCO_3_^-^ were negatively correlated with pH, while HCO_3_^-^ also showed a significant negative correlation with Na^+^. Furthermore, pH, AN, AK, K^+^, Na^+^, Mg²^+^, HCO_3_^-^, Cl^-^, and CEC all exhibited strong and significant correlations with the SQI (r < 0.25, *P* < 0.001).

The application of organic fertilizer resulted in significant positive correlations between the following pairs: HCO_3_^-^ and SOM; Na^+^ and AN; Ca^2+^ and AK; and ESP and ENa (**Figure 8C**, *P* < 0.001). Conversely, AK and Ca^2+^ were significantly negatively correlated with AN and SOM. Additionally, Mg²^+^ and HCO_3_^-^ showed a significant negative correlation with CEC, while Na^+^ was negatively correlated with AK. Multiple parameters, including pH, EC, AN, AP, AK, Na^+^, Ca²^+^, Mg²^+^, HCO_3_^-^, and SAR, were strongly and significantly correlated with the SQI (r < 0.25, *P* < 0.05).

**Figure 9:**
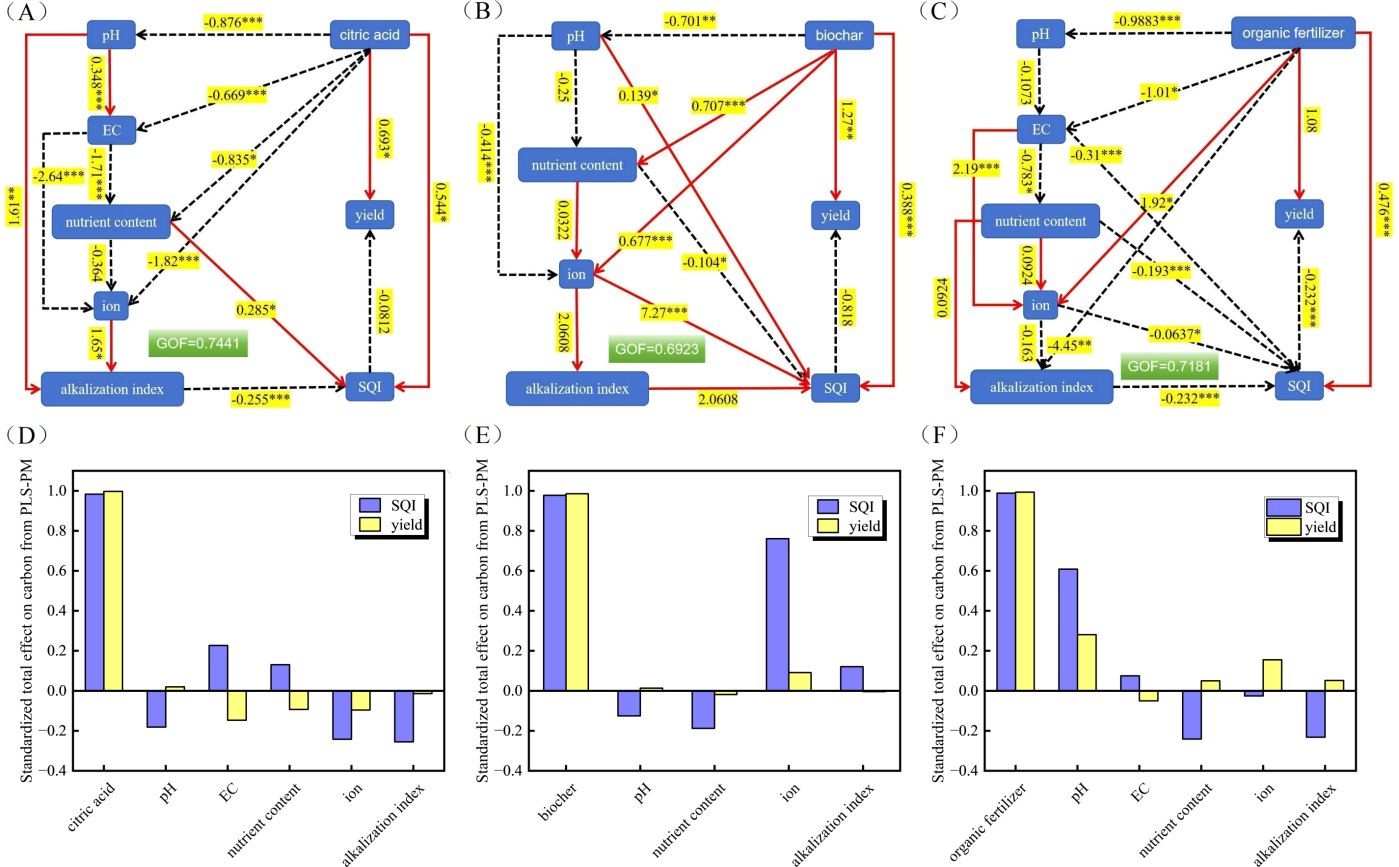
PLS-PM, Partial least squares path modelling of the effects of different amendments on SQI and soybean yield. The overall impact of amendments on soybean yield and the soil quality index. The red solid line in the figure represents a positive effect, and the black dashed line represents a negative effect. *, **, and *** represent *P* < 0.05, *P* < 0.01, and *P* < 0.001, respectively. The adjacent numbers in the same direction as the arrows represent the path coefficient (β). **(A)** Citric acid. The GOF, goodness-of-fit index of PLS-PM model was 0.7441. **(B)** Biochar. The GOF, goodness-of-fit index of PLS-PM model was 0.6923. **(C)** Organic fertilizer. The GOF, goodness-of-fit index of PLS-PM model was 0.7181. **(D)** The standardized total effect of citric acid treatment on SQI and soybean yield. **(E)** The standardized total effect of various parameters of biochar treatment on SQI and soybean yield. **(F)** The standardized total effect of various parameters of organic fertilizer treatment on SQI and soybean yield.

Based on the correlation heatmap results, soil indicators exhibiting a significant influence on the SQI and soybean yield were selected for partial least squares path modelling (PLS-PM). This analysis elucidated the overall effects of the different amendment treatments on both yield and SQI (**Figure 9**). The GOF for the models under different soil amendments treatments was 0.744, 0.692, and 0.718, indicating an acceptable model fit. Furthermore, the PLS-PM results indicated that the factors affecting soybean yield are multifactorial and exhibit complex interactions. The results indicate that citric acid application significantly enhanced soybean yield and the SQI, whereas it reduced soil pH, EC, and the contents of major nutrients and ions (**Figure 9A**). Biochar markedly enhanced soybean yield, alongside nutrient and ion content, and improved the SQI. Conversely, it significantly reduces soil pH (**Figure 9B**). The application of organic fertilizer enhanced soybean yield and exerted a highly significant positive influence on soil ions and the SQI, while concurrently inducing a highly significant negative effect on pH, EC, and soil alkalization parameters (**Figure 9C**). The application of all three soil amendments had a positive effect on soybean yield. The total effect of EC on soybean yield was mainly influenced by the application of citric acid (**Figure 9D**), the standardized total effects were 0.2268, respectively. The total effect of SQI and soybean yield was mainly influenced by the ion content after biochar application (**Figure 9E**), the standardized total effects were 0.7611 and 0.0910, respectively. The total effect of pH on SQI and soybean yield was significantly influenced by the application of organic fertilizer (**Figure 9F**), the standardized total effects were 0.6086 and 0.2809, respectively.

## 4 Discussion

### 4.1 The impact of soil amendments on soil properties

This study employed the SQI to integrate a suite of soil physical and chemical properties, thereby systematically evaluating the comprehensive ameliorative effects of soil amendments on saline-alkali stress and nutrient availability. The results showed that the SQI of citric acid (T1) was highest in the 0–10 cm soil layer. The decay of SQI with increasing soil depth also confirms the shallow nature of citric acid action (Van et al., 2003). Citric acid is a low molecular weight organic acid that is easily mineralized by microorganisms in soil and has poor mobility (Van et al., 2002). Correlation analysis shows that citric acid (T1) enhances SQI by synergistically regulating indicators such as pH, EC, HCO_3_^-^, SAR, AP, AK, K^+^, Ca^2+^. The research results showed that T1 treatment significantly reduced pH and EC. Citric acid promotes salt leaching by dissociating H^+^ and neutralizing OH^-^ and HCO_3_^-^, while chelating with Ca^2+^ and Mg^2+^ (Tang et al., 2021). Research has found that the SAR of citric acid (T1) treatment is significantly lower than that of CK treatment. The carboxyl group of citric acid dissolves insoluble calcium through coordination chelation, releasing Ca^2+^ to replace exchangeable Na^+^ on the colloid, and forming soluble calcium citrate and sodium citrate, promoting Na^+^ leaching with water (Al-Khaldi et al., 2007), leading to a decrease in SAR. The results of this study showed that after treatment with citric acid, the exchangeable sodium content in each soil layer was significantly lower than that of CK treatment, which fully demonstrates the good overall alkali reducing effect of citric acid on soda saline alkali soil. However, exchangeable sodium did not show a unidirectional trend in the profile, but rather showed that the exchangeable sodium content in the 10–20 cm soil layer was higher than that in the 0–10 cm soil layer, while the 20–30 cm soil layer showed a decrease. Low molecular weight organic acid leaching can increase the lower exchangeable base, mainly due to the downward migration of upper base ions (Li and Wang, 2006). In the 20–30 cm soil layer, a decrease in citric acid concentration causes Ca^2+^ to dissociate from the complex and compete for adsorption on the colloidal surface, replacing Na^+^ and being discharged from the soil with the rinsing water, resulting in a further decrease in exchangeable sodium (Miao et al., 2023). Research has shown that citric acid significantly enhances the effectiveness of phosphorus and potassium (Gong and Fan, 2018). This effect is primarily attributed to its capacity to chelate aluminum and iron oxides, forming stable complexes that mobilize fixed phosphorus and potassium (Wang et al., 2024).

In this study, the SQI of each soil layer in biochar (T2) was significantly higher than that in CK treatment. Correlation analysis showed that pH, AN, AK, K^+^, Na^+^, Mg^2+^, HCO_3_^-^, Cl^-^, CEC were significantly correlated with SQI in T2 treatment. The comprehensive improvement of these indicators constitutes the core driving force for T2 to enhance SQI. The experimental results showed that after applying biochar (T2) to saline alkali soil, the soil pH significantly decreased, which is consistent with the research results of Saifullah et al. (2018) and Cai et al. (2024). Biochar can release H^+^ and consume OH^-^ through surface acidic functional groups, leading to a decrease in soil pH and a significant reduction in HCO_3_^-^ content (Zhang et al., 2025). This is consistent with the results of this study. Furthermore, this study identified an inverse relationship between soil pH and electrical conductivity, a finding consistent with the established understanding that these two parameters are negatively correlated (Ruan et al., 2024). Biochar application enhances soil water permeability by increasing porosity and water-holding capacity, thereby promoting salt ion leaching and reducing surface EC (An et al., 2023). However, this process can also lead to the redistribution of salts within the soil profile, subsequently altering the overall EC measurements (Ni et al., 2023). Studies have indicated that biochar with a high ash content can elevate soil soluble salt concentrations following its dissolution, thus enhancing electrical conductivity (Boostani et al., 2023). The active Ca^2+^ and Mg²^+^ carried by biochar replace the exchangeable sodium on the surface of soil colloids into the solution through cation exchange, which is the direct reason for the significant increase in Mg^2+^ content, synchronous decrease in Na^+^ concentration, and synchronous increase in soil CEC (Gao et al., 2023). This study also confirms this conclusion. In addition, the research results showed that the Ca^2+^ in the 0–10 cm soil layer treated with biochar was not significant compared to the CK treatment, while the Ca^2+^ in the 10–20 cm soil layer was significantly reduced compared to the CK treatment, and the Ca^2+^ in the 20–30 cm soil layer was significantly increased compared to the CK treatment. This is because the displaced Ca^2+^ did not accumulate in large quantities in the 0–10 cm soil layer, but quickly leached into the lower layer with the vertical movement of irrigation water, resulting in the residual Ca^2+^ in the 0–10 cm soil layer remaining in a stable state (Kong et al., 2023). In the 10–20 cm soil layer, leaching plays a dominant role. Ca^2+^ and Na^+^ from the upper layer continue to migrate downwards with water, while some Ca^2+^ precipitates within this depth range, resulting in a lower Ca^2+^ content in this layer compared to the CK treatment (Sadegh-Zadeh et al., 2018). In the 20–30 cm soil layer, the HCO_3_^-^ and CO_3_^2-^ contained in the soil solution of this layer precipitate with the Ca^2+^ that migrates here. At the same time, the Ca^2+^ that is not precipitated can be further adsorbed by colloids, resulting in a significantly higher Ca^2+^ content in this layer compared to the CK treatment (Ma et al., 2026). The study found that the exchangeable sodium in each soil layer was significantly lower than that in the CK treatment. Compared with the 0–10 cm soil layer, the exchangeable sodium in the 10–20 cm soil layer continued to decrease, and there was a slight increase in the 20–30 cm soil layer, but it was still lower than that in the 0–10 cm soil layer. In the 10–20 cm soil layer, the continuous leaching of the upper layer drives the downward migration of soluble sodium. Ca^2+^ inhibits the adsorption of Na^+^ and promotes its leaching, resulting in a further decrease in exchangeable sodium compared to the upper layer (Sadegh-Zadeh et al., 2018). After entering the 20–30 cm soil layer, the rate of water infiltration slows down, and the contact time between Na^+^ in the solution and deep colloids is prolonged. In the absence of sufficient competition from multivalent cations, Na^+^ can regain some exchange sites, resulting in a slight increase in exchangeable sodium (Kapoor et al., 1989). Compared with CK treatment, biochar (T2) significantly increased the content of AN, AP, and AK. Zhang et al. (2024) also proposed the same conclusion. Zheng et al. (2018) proposed that biochar (T2) enhances soil nutrient content by adsorbing and fixing functional groups such as surface carboxyl and phenolic hydroxyl groups through a multi-level pore structure, as well as by containing nutrients such as N, P, and K.

The 10–20 cm and 20–30 cm soil layers are dominated by organic fertilizer (T3), which confirms the significant improvement effect of organic amendments on soil quality, consistent with the conclusion of Tian et al. (2024). Correlation analysis showed that pH, EC, AN, AP, AK, Na^+^, Ca^2+^, Mg^2+^, HCO_3_^-^, and SAR were significantly correlated with SQI (*P* < 0.05). Previous studies have established that applying organic fertilizer (T3) effectively lowers the pH of saline-alkali soils through two primary mechanisms (Chen et al., 2021). This is consistent with the results of this study. The low molecular weight organic acids released during the decomposition of organic fertilizers are dissociated by carboxyl groups to produce H^+^, which directly neutralizes CO_3_^2-^ and HCO_3_^-^ in the soil. At the same time, humic acid (HA) in organic fertilizers forms coordination bridges with Ca^2+^ and Mg^2+^ through phenolic hydroxyl groups, blocking the hydrolysis pathway of CO_3_^2-^ to inhibit the generation of OH^-^ and promote soil desalination (Yu et al., 2024; Ruan et al., 2024). The results of this study show that organic fertilizers and citric acid exhibit highly similar behavior in the changes of exchangeable sodium. This is also caused by the large amount of low molecular weight organic acids produced during the decomposition of organic fertilizers. Research has shown that the application of organic fertilizers significantly increases the content of available nitrogen, available phosphorus, and available potassium in soda saline alkali soil. Li et al. (2025) research shows that organic fertilizers are rich in a large number of elements such as nitrogen, phosphorus, and potassium, as well as organic nutrients, which can balance soil nutrient utilization, activate internal soil nutrients, and accelerate the mineralization and decomposition of soil organic matter. Meanwhile, organic fertilizers can enhance soil microbial activity and promote the increase of available nutrients in soil (Qu et al., 2025). This study found that compared with the control (CK), the HCO_3_^-^ content and sodium adsorption ratio (SAR) in the 0–10 cm and 10–20 cm soil layers were significantly reduced under organic fertilizer (T3) treatment, while they showed an increasing trend in the 20–30 cm soil layer. After the application of organic fertilizer, the decomposition of organic acids, neutralization of humus functional groups, and acid production by microbial metabolism are most strongly affected in the surface soil. HCO_3_^-^ is consumed in large quantities, and SAR decreases due to a decrease in Na^+^ and an increase in Ca^2+^ and Mg^2+^ (Wang and Meng, 2022). The application of organic fertilizers can improve soil pore structure and accelerate the leaching and migration of salt ions (Liu et al., 2024). The soluble Na^+^ and incompletely consumed HCO_3_^-^ that are displaced into the soil solution migrate to the 20–30 cm soil layer with the infiltration water flow, forming a secondary accumulation zone in this layer, resulting in a relative increase in HCO_3_^-^ and SAR, indicating that the leaching and redistribution laws of organic materials promoting salt migration from the surface to the deep are consistent (Ni et al., 2023).

### 4.2 The effect of soil amendments on soybean yield

The main goal of improving saline alkali soil is to enhance soil quality, and more importantly, to achieve higher crop yields (Shrivastava and Kumar, 2015). In this study, the application of citric acid, biochar, and organic fertilizer significantly increased soybean yield. This is because the addition of amendments improves soil quality, promotes effective absorption and utilization of nutrients by crops, and thereby increases soybean yield (Liu et al., 2024; Xiao et al., 2024). The results of this study showed that SQI for all layers exhibited a significant positive correlation with both soybean yield and the 100-seed weight (Chang et al., 2023). This finding aligns with the causal mechanism proposed by Zhang et al. (2025), which posits that SQI enhances crop yield by improving the soil’s organic and inorganic fractions. RDA was further employed to quantify the relative contributions of specific soil properties to crop yield (**Figure 6**). Soybean yield and 100-seed weight exhibited a positive correlation with SOM, AN, AP, AK, and CEC, but negatively correlated with pH, EC, ESP, and exchangeable sodium content. Among these factors, ESP was identified as the primary limiting factor in the 0–10 cm layer, whereas pH emerged as the dominant constraint in the 10–20 cm layer. In the deepest layer (20–30 cm), exchangeable sodium content was the principal contributing factor. The observed increase in the SQI is therefore attributable to the synergistic effects of enhanced soil fertility and the alleviation of saline-alkali stress. This finding aligns with and extends the work of Chang et al. (2023), demonstrating that deep soil amendment enhances nutrient cycling capacity within agroecosystems. Mantel tests identified pH, AK, and HCO_3_^-^ as key factors influencing the SQI (**Figure 7**), and based on this, a partial least squares path model (PLS-PM) was constructed.

The study found that PLS-PM revealed a non intuitive path relationship (**Figure 9**). In this model, the direct path coefficient of SQI to yield is negative, which contradicts the analysis results in **Figure 7** that show a positive correlation between SQI and yield. This indicates that the overall positive effect of SQI on yield is actually indirectly achieved through other mediating variables. As a multi index composite index, the increase of SQI does not always translate linearly into yield gains. When the improvement of SQI mainly comes from the decrease of alkalization indicators or the decrease of pH and EC, rather than the synchronous improvement of soil nutrient content, the direct effect of SQI on yield shows a negative characteristic. This also means that the negative effects of salt alkali stress dominate the direct pathway, with their intensity exceeding the contribution of nutrient positive effects. **Figure 8** further confirms this judgment: pH, EC, Na^+^, ESP, SAR and other salt alkali indicators are highly positively correlated with each other and significantly positively correlated with SQI. In summary, the negative correlation between SQI and yield in PLS-PM does not negate the positive effect of the modifier, but rather reveals the limitations of the composite index in nonlinear, multi factor interaction systems. The results of this study emphasize that simply pursuing SQI maximization does not equate to achieving optimal yield; The improvement strategy needs to strike a balance between reducing salt, increasing carbon, preserving fertilizer, and removing sodium.

## 5 Conclusion

The remediation mechanism of citric acid (T1) primarily involves ion-exchange and chemical neutralization. Biochar (T2) functions mainly by introducing a labile carbon source and facilitating physical adsorption. Organic fertilizer (T3) enhances soil nutrient content (AN, AP, and AK) and the SQI through the amendment of highly active organic matter. Among the amendments tested, citric acid demonstrated the greatest efficacy in enhancing the SQI within the surface layer (0–10 cm). In contrast, organic fertilizer was more effective in the subsurface layer (10–30 cm). Furthermore, the SQI across all soil depths exhibited a significant positive correlation with both soybean yield and the 100-seed weight. SOM, nutrient availability, and CEC were positively associated with soybean yield. Conversely, yield is negatively correlated with soil pH, EC, ESP, and exchangeable sodium content. In summary, the application of citric acid, biochar, and organic fertilizer effectively restores the quality of soda saline alkali soil and increases soybean production capacity. Among these treatments, citric acid (T1) was the most effective, due to its ability to rapidly neutralize sodium ions and improve nutrient availability. Consequently, it represents the optimal amendment under the present experimental conditions for achieving simultaneously soil amendment and high-yield soybean production. Different soil amendments not only affect soil nutrients and salinity, but also affect soil biological characteristics. Future research can further explore the effects of different soil amendments on soil biological characteristics to validate their important value in sustainable agriculture.

## Data Availability

The original contributions presented in the study are included in the article/**Supplementary Material**. Further inquiries can be directed to the corresponding author.
